# Computational approaches for investigating interfacial adhesion phenomena of polyimide on silica glass

**DOI:** 10.1038/s41598-017-10994-8

**Published:** 2017-09-05

**Authors:** Kyoungmin Min, Aravind R. Rammohan, Hyo Sug Lee, Jaikwang Shin, Sung Hoon Lee, Sushmit Goyal, Hyunhang Park, John C. Mauro, Ross Stewart, Venkatesh Botu, Hyunbin Kim, Eunseog Cho

**Affiliations:** 1 Platform Technology Lab, Samsung Advanced Institute of Technology, 130 Samsung-ro, Suwon, Gyeonggi-do 443-803 Republic of Korea; 2 Science and Technology Division, Corning Incorporated, One Science Center Drive, Corning, New York, 14831 United States; 3 Corning Technology Center Korea, Asan, Chungcheongnam-do 31454 Republic of Korea

## Abstract

This manuscript provides a comprehensive study of adhesion behavior and its governing mechanisms when polyimide undergoes various modes of detachment from silica glass. Within the framework of steered molecular dynamics, we develop three different adhesion measurement techniques: pulling, peeling, and sliding. Such computational methodologies can be applied to investigate heterogeneous materials with differing interfacial adhesion modes. Here, a novel hybrid potential involving a combination of the INTERFACE force field in conjunction with ReaxFF and including Coulombic and Lennard-Jones interactions is employed to study such interfaces. The studies indicate that the pulling test requires the largest force and the shortest distance to detachment as the interfacial area is separated instantaneously, while the peeling test is observed to exhibit the largest distance for detachment because it separates via line-by-line adhesion. Two kinds of polyimides, aromatic and aliphatic type, are considered to demonstrate the rigidity dependent adhesion properties. The aromatic polyimide, which is more rigid due to the stronger charge transfer complex between chains, requires a greater force but a smaller distance at detachment than the aliphatic polyimide for all of the three methodologies.

## Introduction

Polyimide (PI) materials are recognized for their excellent mechanical properties, thermal stability, and resistivity to chemicals. They are also flexible, lightweight, and electrically insulating, which have made PI a promising material for a wide range of industrial applications^1–6^. For their commercial usage in electronic/electric devices, important considerations about their bonding to other materials arise since they are typically coupled with an adhesive interfacial layer that joins PI with glassy substrates.

The structural integrity at the interface is critical, because the quality of this system largely depends on how this adhesion is managed. For example, in the course of the manufacturing process of flexible displays, PI needs to be attached to the surface of the supporting glass during the stacking process of the display materials^5, 7^. The glass is then detached to complete the process, increasing the probability of structural failure at this interface. There are two possible modes of adhesion failure at the interface, adhesive or cohesive, and controlling the degree of adhesion is important. Preferably, adhesion between the two materials is neither too strong nor too weak. Adhesive failure is observed when the detachment occurs without internal breakage of the two materials, while cohesive failure occurs when the bi-material stack is deformed severely. This cohesive failure causes either the polymer chains to slip and leave residue on the surface of glass, or species such as hydroxyl groups on the glass surface break physically. Within this context, detailed investigation of the interfacial adhesion and its quantification under various conditions are important aspects for enhancing the structural integrity for these systems.

In our previous study, we correlated the thermal stability of aromatic PIs to their adhesion behavior with silica glass^8^. We experimentally measured the coefficient of thermal expansion (CTE) values of various types of PI structures and qualitatively confirmed those with mechanical and structural properties from MD simulations, supporting the rigidity dependent adhesion properties under the pulling test. We further investigated the fundamentals of this relation by looking at the interaction of the polymer chain itself or subunit to the silica with various surface hydroxylation densities^9, 10^. A separate experimental study suggests that no chemical bonding between the PI film and SiO_2_ is observed, indicating that non-bonded interactions are the key factor for understanding their interfacial adhesion^11^. Moreover, it has been demonstrated that the adhesion strength between PI and SiO_2_ can be largely affected by the curing time and also by exposure to UV radiation^12^. In addition to the above-mentioned studies, other important factors have been found significant when SiO_2_ structures interact with different types of polymers. It has been shown that the number of contact/connection points from the polymer (poly acrylic acid based copolymer) chains determine their adhesion to Si surfaces^13^. The degree of humidity also plays a significant role on interfacial properties between epoxy-bonded silica structures^14^.

The measurement of the degree of adhesion can be obtained from numerous methods such as peeling^15, 16^, pulling^17^, bending^18^, sliding^19, 20^, laser spallation^21, 22^, and nano-indentation^23^. These methods only provide basic information about the macroscopic view of deformation at the interface, such as the evolution of the force vs. displacement. However, it is imperative to understand the fundamental aspects of adhesion in greater detail by microscopic exploration of how inter/intramolecular interactions of bi-materials can fundamentally affect the interfacial adhesion properties under the various conditions of the adhesion test modes.

In this regard, the goal of this work is to employ three different types of methodologies for characterizing the interfacial adhesion behaviors of PIs placed on SiO_2_ glass. The pulling, peeling, and sliding methods are implemented within the framework of Steered Molecular Dynamics (SMD) to obtain the full atomistic view of the adhesion phenomena. Two types of PIs (aromatic and aliphatic) are investigated to elucidate their rigidity dependent adhesion properties. The hybridization of interatomic potentials is employed for the description of bi-materials and interfaces. For each of the methods, we quantify global measures of adhesion such as the adhesion force and distance for detachment to compare the three methods. In addition, variations in chain conformation as well as atomic snapshots during each adhesion test are obtained to provide a fundamental understanding on the effect of chain rigidity.

## Materials and Methodology

### Materials

Two different types of PI structures are considered in this study. Their molecular structures are shown in Figure S1(a) in Supplementary Information (SI). An aromatic type PI, biphenyltetracarboxylic dianhydride (BPDA) and 4,4′-oxydianiline (ODA) (denoted as Aro or BPDA-ODA), and an aliphatic PI, 1,2,3,4-butanetetracarboxylic dianhydride (BDA) and 4,4′-diaminodicyclohexylmethane (DMDC) (denoted as Ali or BDA-DMDC) are considered. For the construction of the silica (SiO_2_) glass structure, the initial atomic coordinates are extracted from the Materials Studio (ver. 8.0, Accelrys Inc.) database, whose surface is fully (100%) hydroxylated (OH).

### Steered Molecular Dynamics (SMD) Simulations

Comprehensive understanding of the interfacial adhesion behavior at the atomistic scale can be achieved in greater detail by performing simulations which span large length scales; this mandates the need for large simulations with hundreds of atoms. In this regard, molecular dynamics (MD) is an ideal tool for obtaining the atomic level of examination on intra/inter-system interactions with a reasonable amount of simulation time. The SMD method, which is established on the basis of MD, is a suitable tool for examination of chain dynamics at the interfacial region^24–26^. This method has been widely applied for various organic systems such as polymers, bio-materials, and organic-inorganic interfaces^8, 9, 14, 19, 27–32^.

The theoretical background of the SMD method is as follows. Atoms are connected to a virtual SMD atom via a virtual spring during SMD simulations so that they can be moved when the virtual SMD atom is displaced with a constant velocity (*ν*). For the generation of a potential (*U*), a harmonic spring model is used as

1 $${\rm{U}}=\frac{1}{2}k{[vt-(\vec{r}-{\vec{r}}_{0})\cdot \vec{n}]}^{2}$$

where *k* is the spring constant, *t* is the time, $\vec{r}$ and ${\vec{r}}_{0}$ are the actual and the initial positions of the atoms, and $\vec{n}$ is the direction of pulling. The atoms are allowed to relax during SMD simulations. The force ($\vec{F}$) from the applied velocity is defined as $\vec{F}=-\nabla {\rm{U}}$.

To relate the equilibrium quantity, potential of mean force (PMF), to the non-equilibrium process, the average work of adhesion is calculated based on Jarzynski’s equality^24–26^. The PMF value is computed as

2 $${\rm{PMF}}=-\frac{{\rm{1}}}{\beta }\,\mathrm{log}\langle {e}^{-\beta W}\rangle $$

where *β* = 1/(*k*_B_ *T*) with the Boltzmann constant *k*_B_ and the temperature *T* of the system. *W* is the amount of the work during the pulling test, defined as $W=v\int \vec{F}(t)dt$, and the angular bracket indicates the ensemble average of the specified quantity^33^.

### Interatomic potential

To describe the structural behavior of PI and glassy SiO_2_, the hybridization method of interatomic potentials (INTERFACE force-field + Reactive force-field + Lennard-Jones + Coulombic) is implemented. The INTERFACE force-field (IFF)^34^ and Reactive force-field (ReaxFF)^35–37^ are used for the description of PIs and SiO_2_ glass, respectively and its schematic representation is shown in SI. The interaction at the interface of the materials is described by the 9-6 form of the Lennard-Jones (LJ) potential with a Coulombic (Coul) cutoff of 12 Å. Parameters for ε and σ are obtained from those in ReaxFF. The mixing rule is used for the interaction of a pair of atoms, i.e., σ_ij_ = (σ_ii_ + σ_jj_)/2 and ε_ij = _(ε_ii_∙ε_jj_)^0.5^. The advantages of implementing this hybrid potential is that its computational time is about three times faster than using simulations solely with ReaxFF; hence it can be applied to a much larger system. In addition, since not all elements are currently available for ReaxFF, this hybrid type of potential has greater transferability to various systems. Since the accuracy from the SMD simulation can be dependent upon the changes in the direction and magnitude of the detaching process, investigating the electrostatic interaction and incorporate the changes in the partial charge information between sub-molecules in PI structures and the surface of silica could be critical aspect for improving the accuracy of current model^6^. Nevertheless, it is found that the accuracy of the hybrid potential for the current system is comparable to results from the reactive potential only simulation for the whole structure. We benchmark the potential by validating the PI (BPDA-ODA) chain distribution from the surface of SiO_2_ and the adhesion properties under the pulling test, which we find to be in qualitative agreement with those of the Reactive potential. (Figure S2)

### Simulation methods

For performing MD simulations, initial atomic structures for Aro and Ali PIs are built using the Amorphous Cell generator module in Materials Studio. All of the PIs consist of 20 chains of linear PI, with each chain containing six monomers. Each of the PIs is placed on top of the glassy SiO_2_ so that they are confined in the *z*-direction with the surface area of 82.44 Å × 42.20 Å in the *x*- and *y*-directions with periodic boundary conditions (PBC) along those axes. The thickness of both of the layer structures is around 20 Å. A vacuum distance of 150 Å in the *z*-direction is applied to remove any artificial interaction effect from that direction. MD simulations are then performed using LAMMPS package^38^. The confined structure is relaxed with the NVT ensemble for 1 ns at 300 K, followed by the NPT ensemble for 2 ns at 300 K and 1 atm. To maintain the vacuum in the *z*-direction, the barostat is applied only to the *x*- and *y*-directions. The Nosé-Hoover thermostat and barostat are used for the temperature and pressure control with damping parameters of 100 fs and 1000 fs, respectively. The velocity-Verlet time stepping scheme is applied with a time step of 0.5 fs. Once the structural equilibrium is reached, the adhesion tests of pulling, peeling, and sliding methods are performed using SMD simulations with the NVT ensemble. A constant velocity of 50 m/s and spring constant of 100 kcal/mol/Å^2^ are applied.

## Results and Discussions

### Structural properties

Prior to calculating the adhesion properties between PI and SiO_2_ glass, initial bulk structural properties as well as the mechanical properties for both types of PIs are calculated as shown in Figure S1 and Table SI. It is worth noting that the charge transfer complex (CT-complex) is expected to exist especially for the Aro PI structure because of strong electrostatic attractive forces between electron donor/acceptor in this type of PI^6, 39^ Hence, the chain-to-chain interactions and the chain rigidity in BPDA-ODA could be stronger than those in BDA-DMDC. First, the bulk density of the different PI structures (illustrated in Figure S1(a)) is obtained in Table SI, which is 1.31 and 1.09 g/cm^3^ for BPDA-ODA and BDA-DMDC, respectively. Then, to support the greater rigidity in Aro PI, the stress-strain curves of both PIs are compared as shown in Figure S1(b). Based on this curve, Young’s modulus (*E*) and maximum strength (σ_max_) are also computed. (Table SI). This result supports that BPDA-ODA (*E* = 1.65 GPa, σ_max_ = 418.60 MPa) is stiffer than BDA-DMDC (*E* = 1.54 GPa, σ_max_ = 313.41 MPa).

Additionally, the radius of gyration (*R*_g_) and the end-to-end distance of a chain (*R*_EE_) are calculated to obtain the conformational information of chains. These values can provide a qualitative description for the flexibility of polymer chains from the structural point of view^40, 41^. *R*_g_ is defined as ${R}_{{\rm{g}}}=\sqrt{\frac{1}{N+1}\sum _{i=0}^{N}\langle {({r}_{{\rm{i}}}-{r}_{{\rm{G}}})}^{2}\rangle }$, where ${r}_{{\rm{G}}}=\frac{{\rm{1}}}{N+{\rm{1}}}\sum _{i={\rm{0}}}^{N}{r}_{i}$, *N* is the number of atoms, *r*_*i*_ is the location of the *i* ^th^ atom and *r*_G_ is the location of the center of mass. *R*_EE_ is defined as ${R}_{{\rm{EE}}}=|{r}_{{\rm{N}}}-{r}_{{\rm{0}}}|$, where *r*_N_ and *r*_0_ are the atomic coordinates of both ends in the polymer chain.

The calculated *R*_g_ and *R*_EE_ values and their distributions for each of the chains in both PIs are obtained in Figure S1(c) and (d) and the average values are in Table SI. It is clear that BPDA-ODA has larger values of *R*_g_ and *R*_EE_, indicating that this type of PI is more rigid. This trend of rigidity is also maintained for the slab structure of both PIs as shown in the parenthesis of Table SI. (The distribution of *R*_g_ and *R*_EE_ for this slab structure is shown in Figure S3).

To summarize, the above analysis clearly demonstrates that BPDA-ODA (Aro PI) is more rigid than BDA-DMDC (Ali PI), which is supported by the mechanical property and chain conformation. The information on chain conformation is especially useful to characterize the rigidity dependent response in each PI during adhesion tests, which will be discussed later in Section **3.4**.

### Measuring interfacial adhesion properties: Pulling, Peeling, and Sliding methods

In general, computing the binding energy provides the fundamental information on the magnitude of adhesion at the interface of bi-materials such as polymer/glass, dielectric materials/semiconductor, and organic molecules/substrates. Despite its simplicity, it cannot predict dynamic properties such as the morphology changes during a detachment process nor how the interfacial properties change with respect to applied deformation and time. In particular for polymer/glass systems, since the polymer chains could untangle, slip, or deform under external factors, the adhesion behavior can be dependent upon the direction and magnitude of the detaching process, chain rigidity, and binding nature at the interface.

**Figure 1:**
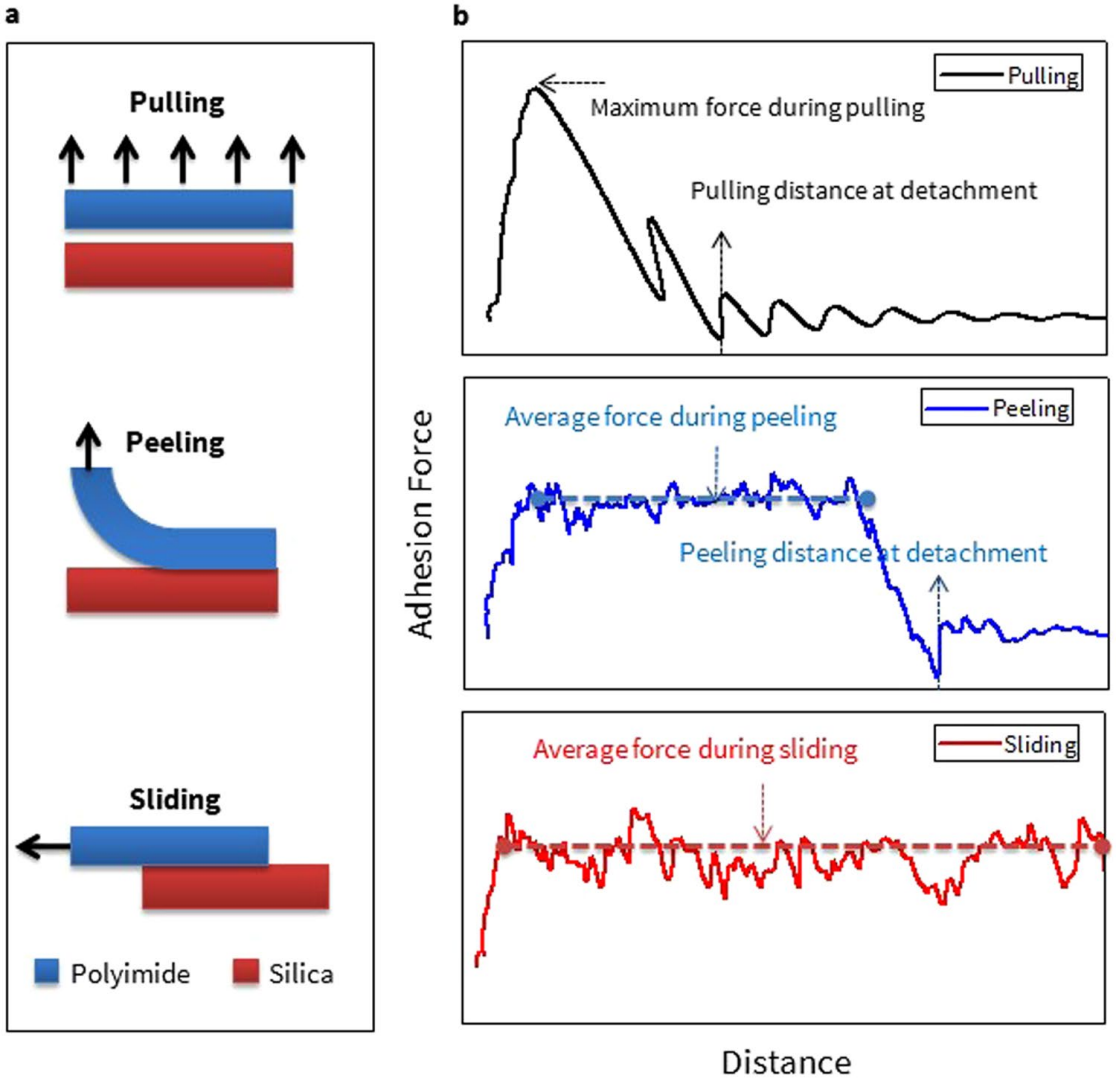
(**a**) The schematic view and (**b**) representative properties for characterizing the adhesion properties from each of adhesion testing modes.

To address the above issues regarding interfacial adhesion behavior, here we implement three methodologies for characterization of the adhesion properties at the PI/glass interface: pulling, peeling, and sliding tests. In Fig. 1(a), we describe the schematic view of how each of the methods is realized. It is important to note that they are fundamentally different modes depending on how the SMD deformation is applied with respect to the interfacial area and the loading direction on the bi-materials. For example, in the case of a pulling test, a uniform velocity is applied to the outward direction for all atoms in the PI and silica, respectively. Employing the peeling method is the same as pulling except that the velocity is applied to a region which is smaller than that of pulling. For modeling the sliding mode, a uniform sliding velocity is applied to the PI atoms and the silica for the in-plane (slip) direction.

In order to understand the adhesion behaviors between PI and silica glass under various detachment modes, it is critical to introduce properties which can properly depict the interfacial region and characterize each of the adhesion testing methods. For this purpose, two descriptive adhesion properties are suggested in Fig. 1(b): the adhesion force and distance to complete detachment. It is important to analyze two different types of forces depending on the methods: the maximum force from pulling (${{\rm{F}}}_{{\rm{Max}}}^{{\rm{Pulling}}}$) and the average force from peeling (${{\rm{F}}}_{{\rm{Avg}}}^{{\rm{Peeling}}}$) and sliding (${{\rm{F}}}_{{\rm{Avg}}}^{{\rm{Sliding}}}$) methods. The distance at detachment can be obtained only from pulling (${{\rm{D}}}_{{\rm{Pulling}}}$) and peeling (${{\rm{D}}}_{{\rm{Peeling}}}$). This value is obtained from the distance between the centers-of-mass of the regions where the velocity is applied in PIs and SiO_2_ when they are fully separated. However, due to PBC effect in the sliding direction, the interface of the bi-materials is conserved (they remain in contact) during sliding so this distance cannot be defined for this case. A detailed process of how each methodology is implemented will be discussed in the following section. For establishing three methods properly at first, Aro PI (BPDA-ODA) is investigated.

#### Pulling vs. Peeling

**Figure 2:**
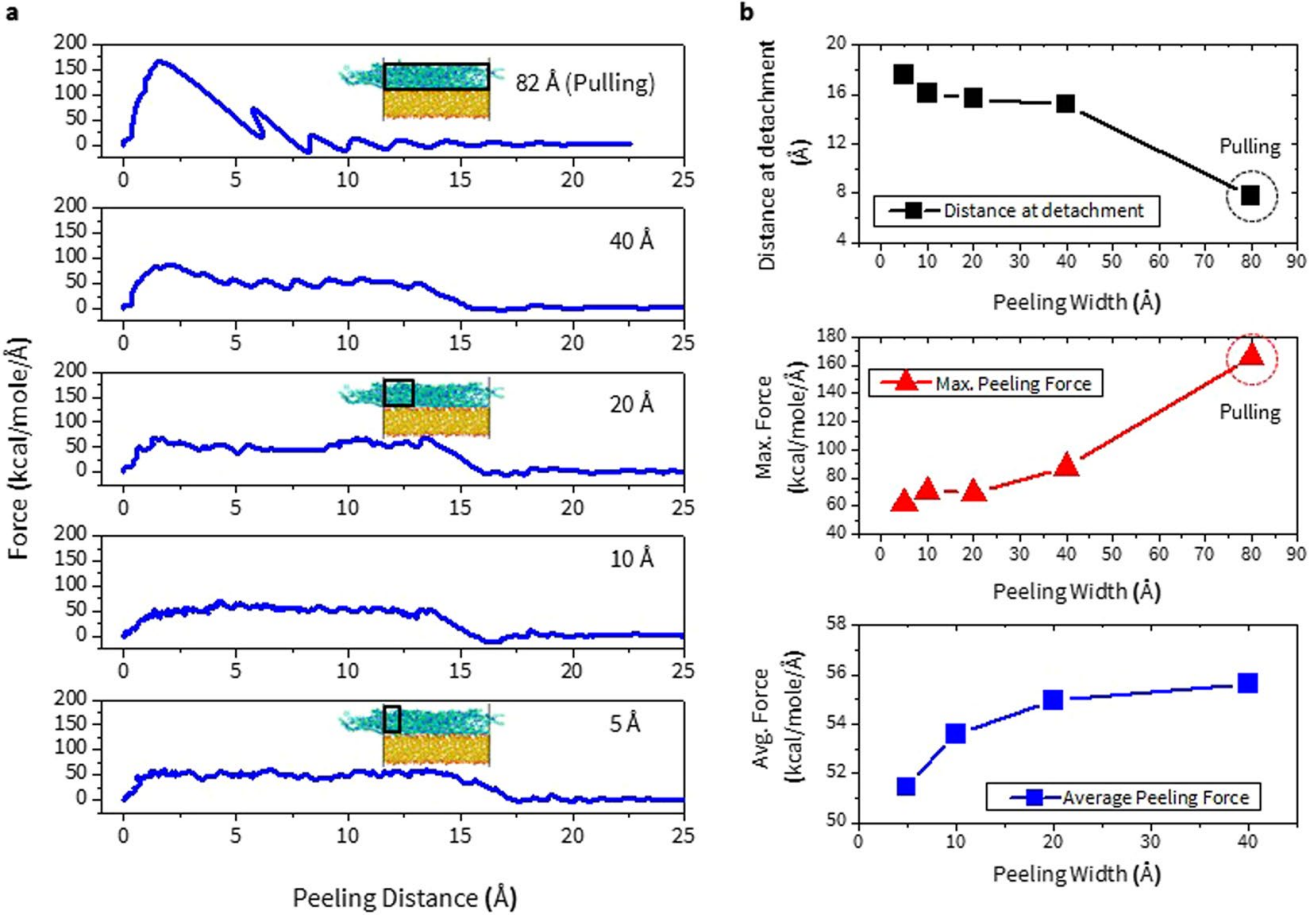
For BPDA-ODA on silica glass, (**a**) Force vs. peeling distance and (**b**) distance at detachment, maximum force, and average force as a function of peeling width (5 Å to 82 Å, pulling). The black box represents the area where peeling force is applied.

As discussed earlier, the peeling method is in principle similar to the way in which the pulling method is implemented in terms of its steered direction. The key difference is that the area where the peeling velocity is applied is smaller; in other words, when one enlarges the peeling-velocity applied region, adhesion properties will start to become comparable to those from pulling. As shown in Fig. 2(a), when the peeling width is increased from 5 Å to 82 Å (pulling) in the *x*-direction (the length in the *y*-direction is constant as 42.20 Å), the plateau region in the response of the peeling force disappears after 40 Å of width. The point where maximum force is obtained starts to emerge around the peeling distance of 2 Å and at a peeling width of 20 Å. Obtaining the plateau region of peeling force is important because this indicates that the adhesion between PI and SiO_2_ glass is homogeneous, so that the average value in that region can be used to represent the interfacial strength in bi-materials.

From our previous study under the pulling test, the adhesion energy, defined as the maximum PMF value divided by the projected interfacial area of PI on SiO_2_, is obtained^8, 9^. Then its convergence behavior with respect to increasing pulling velocity is calculated to find the optimal pulling velocity. However, for the peeling case, the convergence of the adhesion energy is difficult to achieve because the PMF can be largely pre-determined from the peeling width. This is expected because the longer peeling width leads to shorter distance at full detachment, resulting in smaller PMF. (Figure S4).

Accordingly, in Fig. 2(b), the other types of adhesion properties, distance at complete detachment (${{\rm{D}}}_{{\rm{Pulling}}}$ and ${{\rm{D}}}_{{\rm{Peeling}}}$), maximum and average force (${{\rm{F}}}_{{\rm{Max}}}^{{\rm{Pulling}}}$, ${{\rm{F}}}_{{\rm{Max}}}^{{\rm{Peeling}}}$, and ${{\rm{F}}}_{{\rm{Avg}}}^{{\rm{Peeling}}}$) are obtained with respect to the peeling width for finding the optimal parameters. This figure shows that as the peeling width increases, ${{\rm{D}}}_{{\rm{Peeling}}}$ decreases slightly up to 40 Å of the width beyond which the drop becomes abrupt. Meanwhile, ${{\rm{F}}}_{{\rm{Max}}}^{{\rm{Peeling}}}$ becomes larger with increasing width, and the increase is significant after 20 Å of the width, indicating that the peeling width should be less than this value. The overall variation of the average force value (${{\rm{F}}}_{{\rm{Avg}}}^{{\rm{Peeling}}}$), around 4 kcal/mole/Å, is relatively small compared to the other properties and after 20 Å of the peeling width; it then reaches a plateau-like region. Based on the above observations, the peeling width of 20 Å is chosen as an optimal parameter to represent the peeling response and this value is used later in this work for the comparative study of adhesion behaviors in Aro and Ali PI on silica glass.

#### Sliding

**Figure 3:**
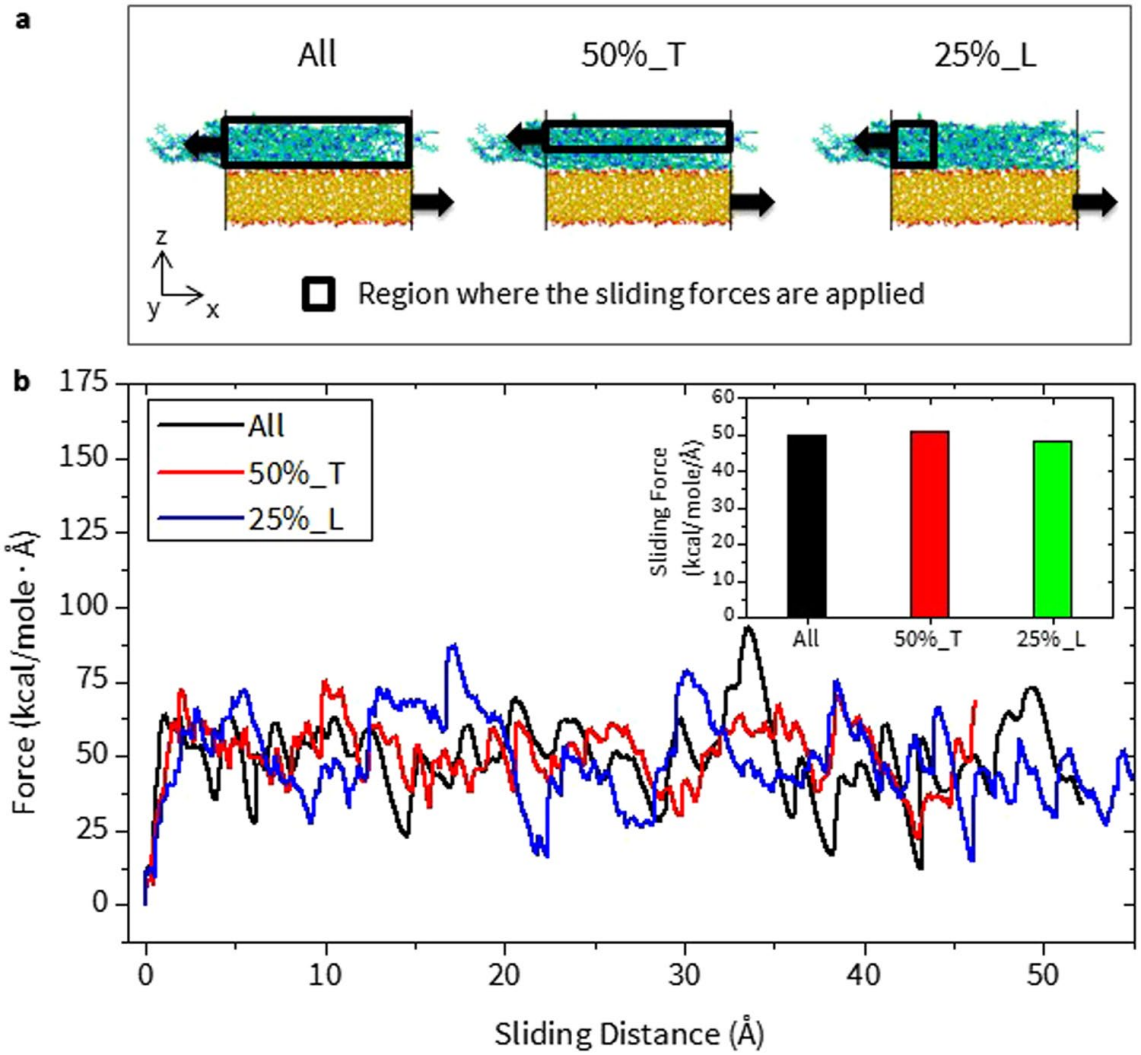
(**a**) The schematic view of the sliding setup for determining an ideal region where the sliding force should be applied. (**b**) Sliding force vs. distance curve for all three cases.) (Inset) The average sliding force for each setup. 50%_T and 25%_L indicates that the peeling force is applied to 50% of the total thickness in z-direction and 25% of the total length in x-direction of the PI.

For the case of sliding, the region where the velocity is applied could also determine the sliding response and thus the adhesion properties. As shown in Fig. 3(a), three different types of sliding tests are examined when the sliding velocity is applied to (a) all of the atoms (All), (b) 50% of the PI thickness in the *z*-direction and 100% of the PI length in the *x*- and *y*-direction (50%_T), and (c) 25% of the PI length in the *x*-direction and 100% of the PI thickness in the *z*-direction and the PI length in the *y*-direction (25%_L). It is important to mention that from all cases, the responses of sliding force vs. distance are hardly discernible from one another. (Fig. 3(b)) More importantly, the average sliding force value of each case, ${{\rm{F}}}_{{\rm{Avg}}}^{{\rm{Sliding}}}$, are almost the same. (Inset of Fig. 3(b)) Although the sliding force plateaus during the test, minimal fluctuations in the response are observed for the 50%_T case. (The standard deviation of the force variation is seen to be 9.83, 13.09, and 14.21 kcal/mole/Å for the case 50%T, 25%_L, and All, respectively.) Based on this, the 50%_T case is chosen for representing the sliding method and used later for a comparative study of Aro and Ali PIs.

### Rate dependent force responses

**Figure 4:**
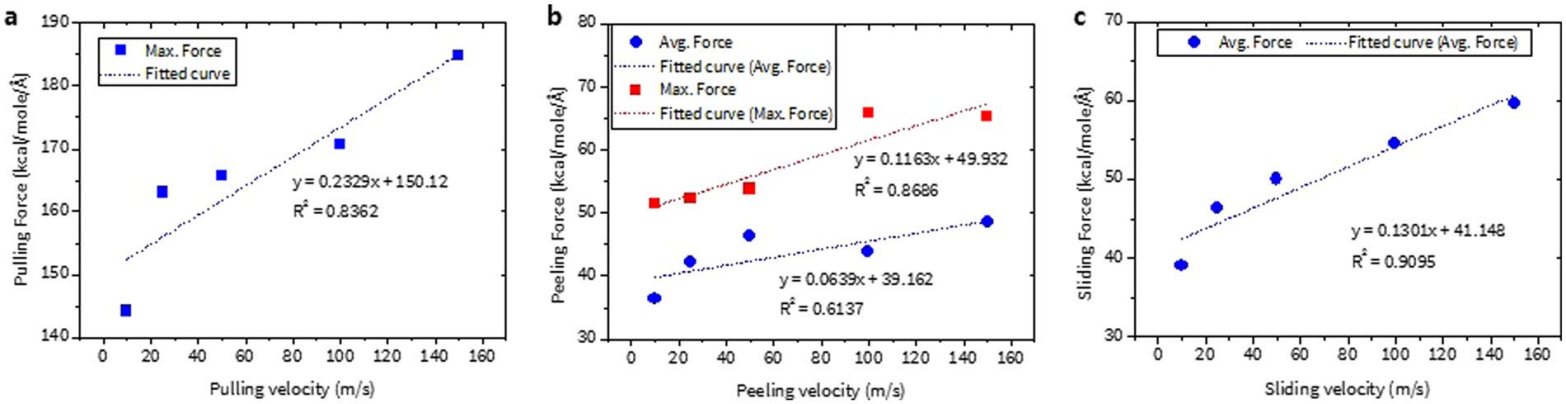
Velocity dependent adhesion force under (**a**) pulling, (**b**) peeling, and (**c**) sliding test.

The adhesion forces obtained from each of the three different methods depend on how fast the velocity is applied because a faster velocity means that a larger force is required to recover the SMD atoms to the virtual SMD atom position^24, 26^. To investigate this dependence, the force vs. velocity relations for all of the methods are obtained in Fig. 4 when an applied velocity is increased from 10 m/s to 150 m/s.

As expected, the force values from all cases increase as applied velocity becomes larger. However, the slope of force (i.e., the sensitivity) exhibits distinctive behavior depending on which method is implemented. The slope for ${{\rm{F}}}_{{\rm{Max}}}^{{\rm{Pulling}}}$, ${{\rm{F}}}_{{\rm{Avg}}}^{{\rm{Peeling}}}$, and ${{\rm{F}}}_{{\rm{Avg}}}^{{\rm{Sliding}}}$ shows that the pulling case exhibits the largest value of 0.2329, followed by the sliding (0.1301) and the peeling force is the least sensitive (0.0639). Meanwhile, it is important to note that for the case of peeling, the slope of ${{\rm{F}}}_{{\rm{Max}}}^{{\rm{Peeling}}}$ is slightly larger (0.1163) than that of ${{\rm{F}}}_{{\rm{Avg}}}^{{\rm{Peeling}}}$ but both values are still smaller than the others. (Fig. 4(b)) (Note that ${{\rm{F}}}_{{\rm{Max}}}^{{\rm{Peeling}}}$ appears at the initial stage of detachment.)

Overall, this result indicates that a much greater force is required to pull off or slide away the polymer. Another finding is that based on the convergence of force values with respect to the applied velocities, a velocity of 50 m/s is chosen and used for the rest of the studies. Although using a smaller value of velocity would be more appropriate to mimic the experimental environment, using this value could be an ideal option for the following reasons. a) The increase of ${{\rm{F}}}_{{\rm{Max}}}^{{\rm{Pulling}}}$ is small within the range of 25 m/s to 100 m/s. b) ${{\rm{F}}}_{{\rm{Avg}}}^{{\rm{Peeling}}}$ value is almost converged after 50 m/s. c) The increase of ${{\rm{F}}}_{{\rm{Avg}}}^{{\rm{Sliding}}}$ up to 50 m/s is rapid but then reduces afterwards. (Fig. 4(c))

### Adhesion properties for aromatic vs. aliphatic PI on silica glass

**Figure 5:**
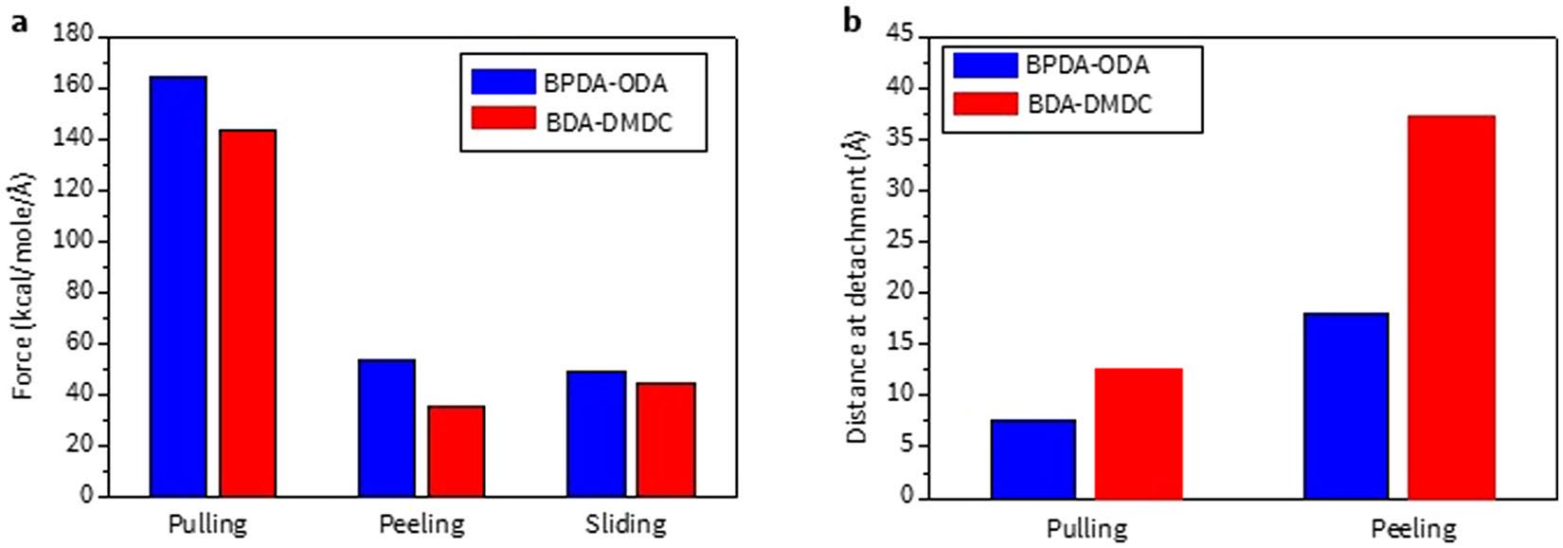
The overview of (**a**) adhesion force and (**b**) distance at detachment for each of adhesion testing methods.

Figure 5 shows an overall comparison of the adhesion properties of two types of PIs on silica glass from using three different methodologies. First, the pulling test requires the largest force for detachment while force values from the peeling and sliding test are more comparable. However, in terms of the distance at complete detachment, a longer distance is necessary for peeling than for the pulling method.

The aforementioned observations could be intuitively understood as follows. In the case of pulling, the pulling velocity is applied to all atoms for detachment of the whole interfacial area at once; thus a larger force should be required. For the peeling test, however, it detaches the PI from the surface of silica in a gradual manner (line by line separation) hence less force is necessary for peeling off the PI but a much longer distance for its complete detachment.

Another important aspect that should be addressed is how the different type of PI can affect the adhesion behavior. For both the pulling and peeling tests, Aro PI (BPDA-ODA) requires a larger force while the distance at detachment is longer from the Ali PI (BDA-DMDC). The governing mechanism of this response could be the rigidity difference between these PIs. Since Ali PI is less rigid than Aro PI, more conformational variation in chains is expected during the detachment process, which can affect the adhesion properties at the interface extensively. A detailed analysis of each method is presented in the following sections.

#### Pulling test

**Figure 6:**
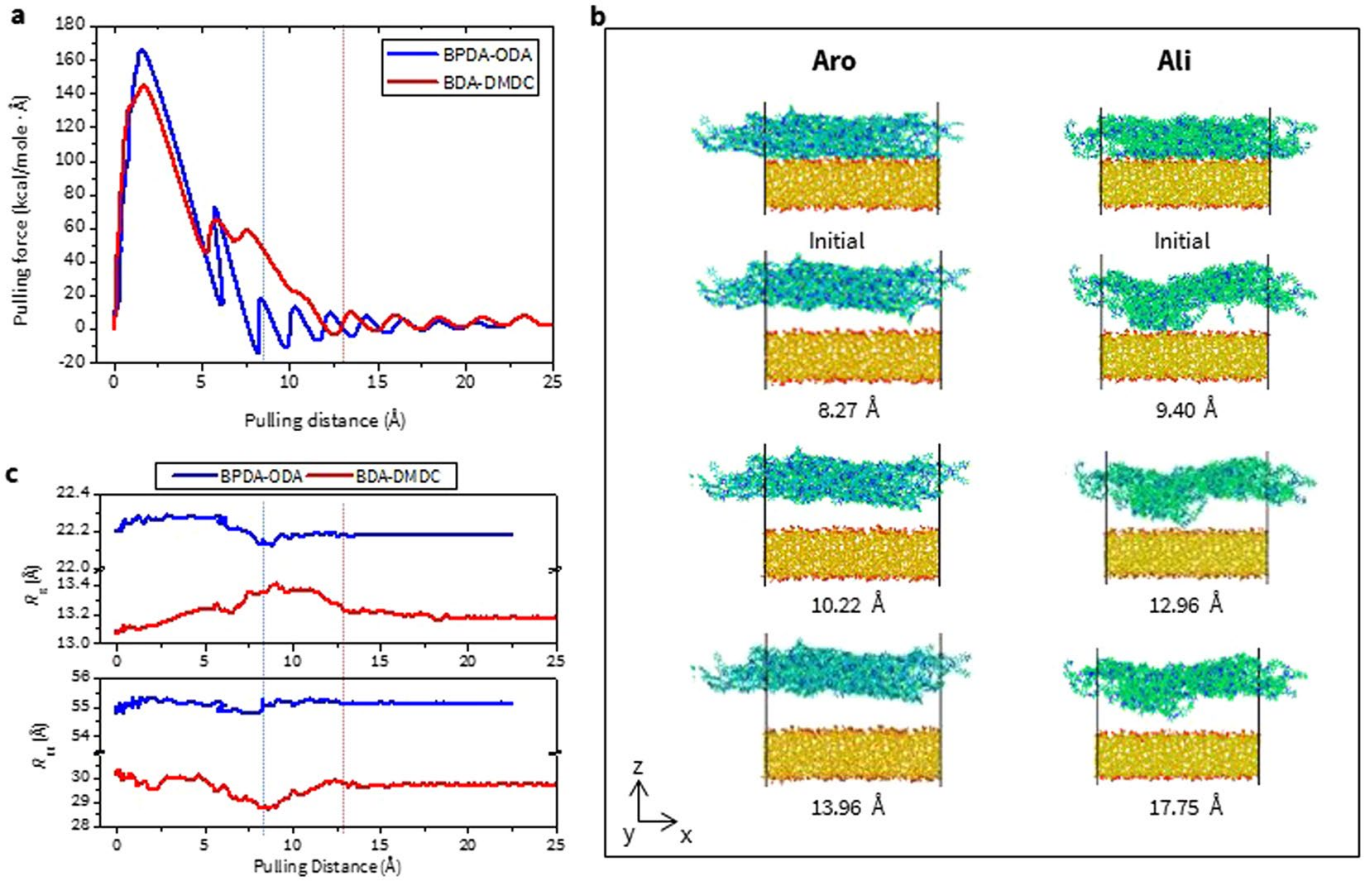
(**a**) Pulling force vs distance, (**b**) snapshots of atomic configuration, (**c**) R_g_ and R_EE_ during pulling test for BPDA-ODA and BDA-DMDC on silica glass, respectively. Blue and red line indicates where the detachment occurs. Numbers in the distance unit represents the change of center-of-mass distance between two materials with respect to their initial position.

Figure 6(a) shows the pulling force vs. distance curve for the structure of BPDA-ODA and BDA-DMDC on SiO_2_, respectively. It clearly indicates that BPDA-ODA requires a larger value of ${{\rm{F}}}_{{\rm{Max}}}^{{\rm{Pulling}}}$ but a shorter ${{\rm{D}}}_{{\rm{Pulling}}}$. For this reason, the adhesion energy (PMF/surface area) from two PIs is almost comparable with each other as shown in Figure S5 (0.208 and 0.226 kcal/mol/Å^2^ for Aro and Ali, respectively). As discussed in our previous work, polymer chains near the surface could play an important role in determining the chain morphology change and inherent adhesion properties^8^. Thus, in order to obtain a view of the pulling process at the atomistic level, snapshots are presented in Fig. 6(b) that are associated with the pulling behavior for each of PIs.

First, since the large interfacial area of bi-materials need to be detached promptly during the pulling process, the amount of force rapidly increases and reaches the maximum as discussed above, which can be inferred from the atomistic snapshots as well. For BPDA-ODA case, ${{\rm{F}}}_{{\rm{Max}}}^{{\rm{Pulling}}}$ is obtained around 2 Å of the pulling distance, and it starts to drop rapidly. (Fig. 6(a) **)** Then it is already fully detached after the pulling distance of 8 Å; thus the chain morphology is almost unaffected during further pulling compared to the initial structure. (Fig. 6(b) **)** Similarly for BDA-DMDC, ${{\rm{F}}}_{{\rm{Max}}}^{{\rm{Pulling}}}$ occurs around 2 Å of pulling distance. However, the decrease in force is smaller than with BPDA-ODA. This is because unlike Aro PI, where the interfacial surface is detached at once, the detached surface of Ali PI is around 50% at the pulling distance of 9.40 Å (2^nd^ row in Fig. 6(b)) and its corresponding force is around 50 kcal/mol/Å. In addition, during further pulling process, polymer chains located closer to the surface are still attached and they start to stretch (3^rd^ row) in the out-of-plane direction, leading to significant changes in the chain conformation. As a result, even after full detachment, the chain nearer to the surface is still deformed contrary to BPDA-ODA whose bottom surface is flat and smooth (4^th^ row).

The quantitative analysis of the above-mentioned observations can be achieved by calculating the variation of *R*_g_ and *R*_EE_ values during the pulling test as shown in Fig. 6(c). This information can provide the overall view of how polymer chains in each type of PI behave differently. As expected for BPDA-ODA, its *R*_g_ (−0.175 Å) and *R*_EE_ ( + 0.597 Å) values exhibit minor change up to full detachment (blue dashed line), which is agreeable with what is observed from the atomic snapshots. On the contrary, a significant variation in *R*_g_ ( + 0.344 Å) and *R*_EE_ (−1.698 Å) values is obtained for BDA-DMDC, which is attributed to the deformed chains closer to the surface. To summarize, the overall results in Fig. 6 fully support our hypothesis that the rigidity difference between PIs, which originates from their intrinsic chain characteristics, can significantly affect the adhesion properties.

#### Peeling test

**Figure 7:**
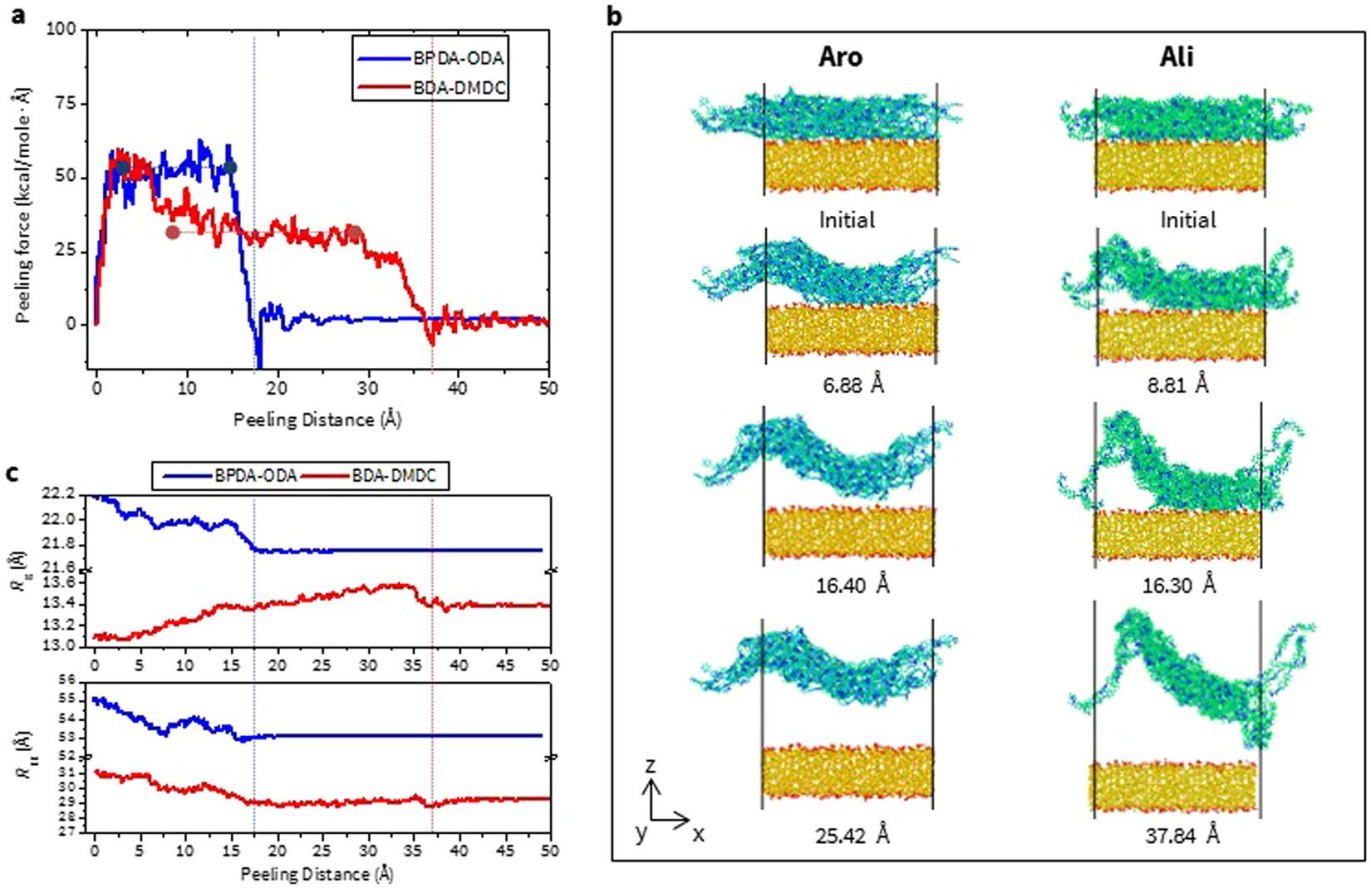
(**a**) Peeling force vs distance, (**b**) snapshots of atomic configuration, (**c**) R_g_ and R_EE_ during pulling test for BPDA-ODA and BDA-DMDC on silica glass, respectively. Blue and red line indicates where the detachment occurs. Numbers in the distance unit represents the change of center-of-mass distance between two materials with respect to their initial position.

The peeling force vs. distance curves for BPDA-ODA and BDA-DMDC on SiO_2_ glass are shown in Fig. 7(a). As expected, a larger value of ${{\rm{F}}}_{{\rm{Avg}}}^{{\rm{Peeling}}}$ (53.58 vs. 35.68 kcal/mole/Å) but a shorter ${{\rm{D}}}_{{\rm{Peeling}}}$ (17.98 vs. 37.15 Å) is required for BPDA-ODA compared to BDA-DMDC. In addition, it is interesting to note that ${{\rm{F}}}_{{\rm{Max}}}^{{\rm{Peeling}}}$ values for both of the PIs are quite comparable (63.17 and 59.71 kcal/mol/Å for Aro and Ali, respectively). A slightly larger value of ${{\rm{F}}}_{{\rm{Max}}}^{{\rm{Peeling}}}$ obtained from Aro PI could be attributed to the fact that the interfacial area needs to be detached at the initial stage of peeling, which is similar to the pulling case. Hence, the initial peeling response is from the area detachment instead of the line separation so one should disregard this period. Soon after this point where ${{\rm{F}}}_{{\rm{Max}}}^{{\rm{Peeling}}}$ is obtained, the peeling force gradually decreases and reaches the plateau region, which properly represents the adhesion properties during the peeling process.

Detailed insights of how the applied peeling force affects the conformational variations of PI chains can be obtained from the atomistic snapshots during the peeling process. (Fig. 7(b)) Unlike the pulling test, the peeling method generally induces a larger deformation in chains. This behavior is obvious since the peeling force is applied over a much smaller region (25%) of PI comparing to that in the pulling case (100%), so the force is localized only in that area. Thus, those regions need to be detached first while the remaining regions are still on the surface of SiO_2_ at the beginning of the peeling process. In addition, due to periodicity in the *x*- and *y*-directions of the simulation box, the PI structure on the right side can also be detached simultaneously during the initial period of peeling. For this reason, PI chains experience more substantial changes in their conformation compared to pulling. This is why obtaining a plateau region in the peeling force vs. distance curve is critical to investigate the interfacial adhesion behavior during the peeling process.

The comparison between conformational changes in Aro and Ali structures clearly indicate that more deformation is observed with Ali. Once peeling force is applied (2^nd^ row in Fig. 7(b)), the initial chain deformation is similar to each other but further peeling induces significantly different responses in each of the PIs. For example, at full detachment of the Aro structure (3^rd^ row in Fig. 7(b)), the sinusoidal shape of PI is obtained and its amplitude becomes slightly reduced due to the chain relaxation effect during further peeling. On the contrary, the fragment of the Ali structure on the right side is still attached even when a part of the PI chains are already detached. More importantly, it is observed that some of the chains are almost untangled, which can be attributed to the lower rigidity of the Ali structure. Finally, when the Ali PI is completely detached (4^th^ row), we see a much more deformed shape and some parts of the PI chain seem to be dragged to the surface of silica, which plays a significant role of a larger D_Peeling_ value.

Lastly, the variation of *R*_g_ and *R*_EE_ values from both PIs during the peeling test is obtained in Fig. 7(c) to quantitatively explain the behavior of deforming chains. As expected, BDA-DMDC experiences a larger variation in *R*_g_ and *R*_EE_ (each value is + 0.530 Å and –2.512 Å, respectively) while −0.463 Å and −1.871 Å are obtained from BPDA-ODA, respectively. The increasing behavior (positive) of *R*_g_ in the Ali structure seems to be associated with the untangled chains observed during peeling. It is also important to note that since the peeling test induces more chain morphological changes than pulling, a larger value of *R*_g_ and *R*_EE_ variations are obtained from the peeling test.

#### Sliding test

**Figure 8:**
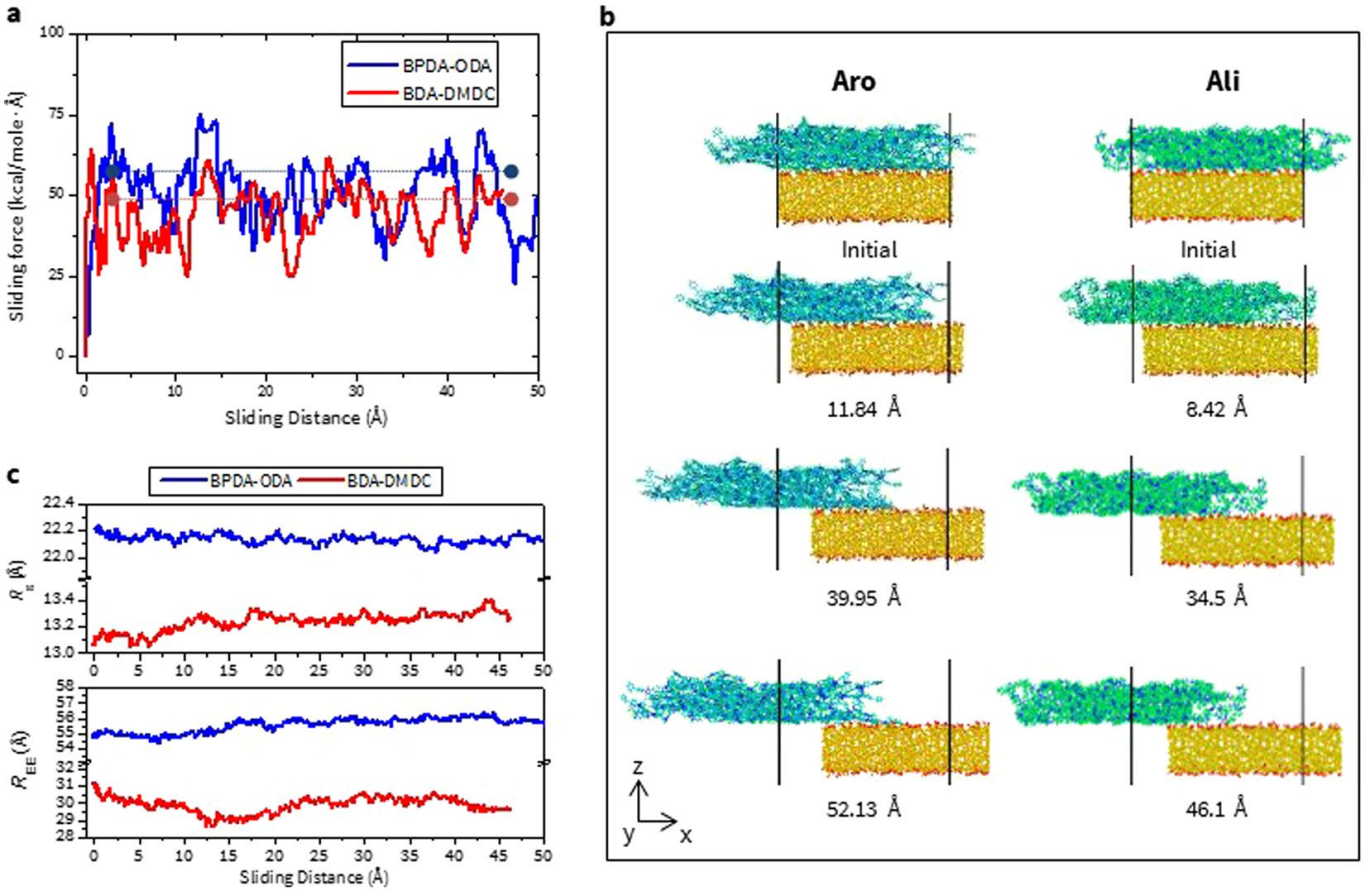
(**a**) Sliding force vs distance, (**b**) snapshots of atomic configuration, (**c**) R_g_ and R_EE_ during pulling test for BPDA-ODA and BDA-DMDC on silica glass, respectively. Numbers in the distance unit represents the change of center-of-mass distance between two materials with respect to their initial position.

Finally, the sliding force vs. distance curve for BPDA-ODA and BDA-DMDC on SiO_2_ glass is presented in Fig. 8(a). This figure shows that a slightly larger value of ${{\rm{F}}}_{{\rm{Avg}}}^{{\rm{Sliding}}}$ (49.66 kcal/mol/Å) is obtained for BPDA-ODA than for BDA-DMDC (44.41 kcal/mol/Å). However qualitatively, the overall response is similar to each other, which can be explained from the atomistic snapshots in Fig. 8(b). It clearly depicts that during the sliding process, the conformation of chains of both PIs is preserved close to their initial structures, which is in contrast to those observed during the pulling or peeling processes. This behavior can be explained quantitatively by *R*_g_ and *R*_EE_ variations in Fig. 8(c). Since detachment does not occur for sliding case, these values are obtained from the absolute value of overall changes during the sliding process. First, their values are generally smaller than those from other methods, indicating that chain conformation is not significantly affected during the sliding process. *R*_g_ and *R*_EE_ of BPDA-ODA are calculated as 0.139 Å and 1.491 Å, respectively, and from BDA-DMDC, they are 0.334 Å and 1.589 Å, respectively. Again, their values are observed to be larger in the Ali structure, further supporting the role of rigidity on determining adhesion properties. This behavior originates from a slightly greater conformational change in both edges of BDA-DMDC during sliding.

## Conclusions

In this study, we characterize the adhesion properties under various types of detachment modes to provide fundamental insights into the interfacial adhesion phenomena for PIs on the SiO_2_ glass structure. The hybridization of interatomic potential (ReaxFF + INTERFACE FF + LJ/Coul interaction) has been successfully applied for description of PIs, SiO_2_ glass, and interfacial interactions. For adhesion measurement, three different methods are employed within the framework of SMD simulation: pulling, peeling, and sliding. The simulations demonstrate that the pulling test exhibits the strongest dependence on the pulling rate while the peeling test is the least sensitive. It is also found that the pulling test requires the largest force because the interfacial area needs to be detached instantaneously. Furthermore, the farthest distance at detachment is observed for peeling because it results in line by line separation. In addition, Aro (BPDA-ODA) and Ali (BDA-DMDC) type of PIs are described to investigate the dependence of adhesion properties on polymer rigidity. Initial bulk properties such as density, mechanical properties, and chain morphologies are calculated, and these results show consistency for description of chain rigidity. The three methodologies consistently demonstrate that the more rigid PI (BPDA-ODA) requires a larger force but a shorter distance for detaching it from silica glass. A detailed analysis of the chain conformation changes further supports the conclusion that BDA-DMDC experiences a larger conformational change and subsequently more variation of *R*_g_ and *R*_EE_ values due to its lower chain rigidity. We believe that current findings and developed methodologies could be applied widely to characterize and improve the structural reliability for hybrid/composites materials such as the manufacturing process of the flexible display and the anti-fingerprint coating on the mobile devices, whose adhesion behaviors at the interface are largely affected from different deformation modes.

## Data availability

The datasets generated during and/or analysed during the current study are available from the corresponding author on reasonable request.

## Electronic supplementary material

Supplementary Information
