# Supplementary material for: Computational approaches for investigating interfacial adhesion phenomena of polyimide on silica glass
Source: Sci Rep. 2017 Sep 5;7:10475. doi: 10.1038/s41598-017-10994-8 (PMC5585183; doi:10.1038/s41598-017-10994-8)
Supplement: Supplementary file 1 — Supplementary Information [file 41598_2017_10994_MOESM1_ESM.pdf]

Supplementary Information

Computational approaches for investigating interfacial adhesion phenomena of polyimide on silica glass

Kyoungmin Min[1], Aravind R. Rammohan[2], Hyo Sug Lee[1], Jaikwang Shin[1], Sung Hoon Lee[3], Sushmit Goyal[2], Hyunhang Park[3], John C. Mauro[2], Ross Stewart[2], Venkatesh Botu[2], Hyunbin Kim[3], and Eunseog Cho[1,*]

[1]Platform Technology Lab, Samsung Advanced Institute of Technology, 130 Samsung-ro, Suwon, Gyeonggi-do, 443-803, Republic of Korea
[2]Science and Technology Division, Corning Incorporated, One Science Center Drive, Corning, New York 14831, United States
[3]Corning Technology Center Korea, Asan, Chungcheongnam-do, 31454, Republic of Korea

*Corresponding Author, E-mail: eunseog.cho@samsung.com (E. Cho)

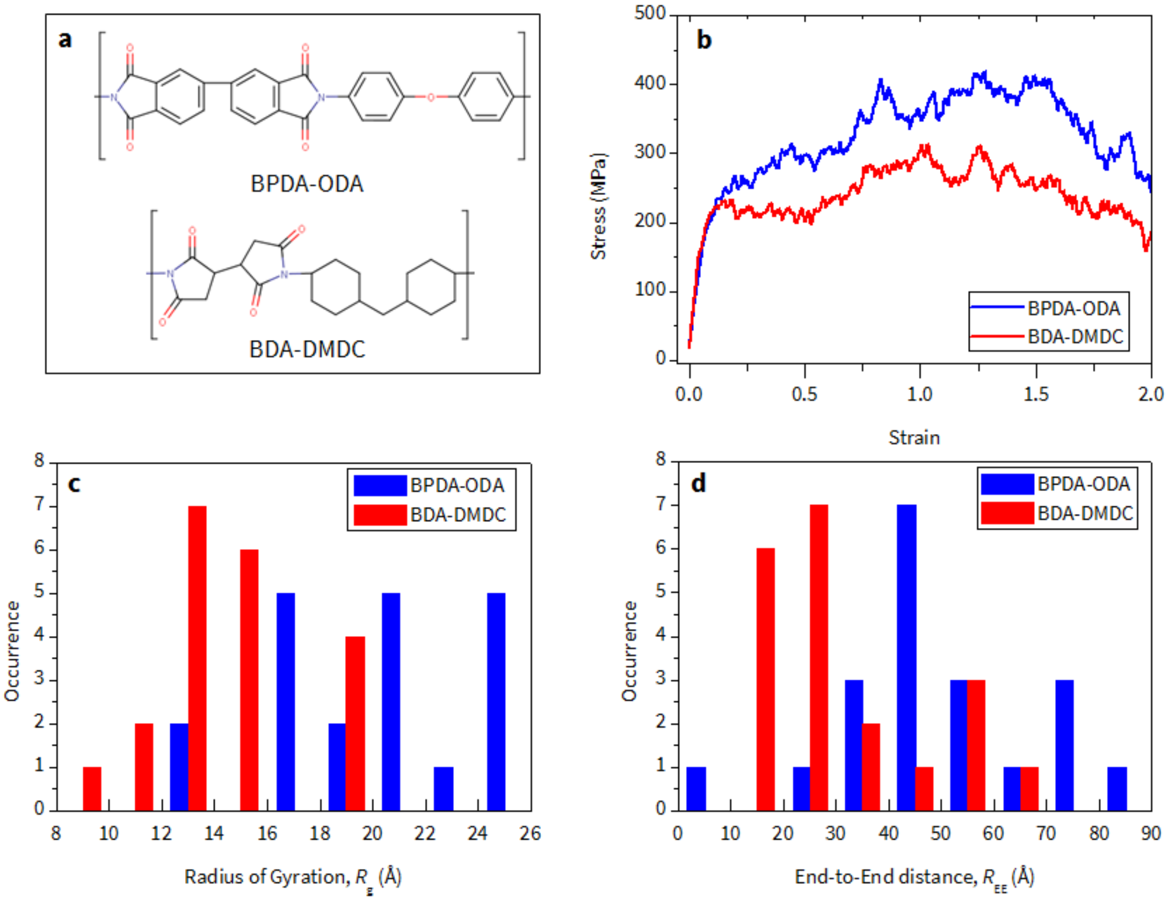

Figure S1. (a)The molecular structure, (b) stress-strain curve under uni-axial tensile test, (c) R_g, and (d) R_{EE} distribution of bulk types of BPDA-ODA and BDA-DMDC, respectively.

	BPDA-ODA	BDA-DMDC
Density [g/cm^3]	1.31 ± 0.01	1.09 ± 0.01
Elastic modulus [GPa]	1.65 ± 0.12	1.54 ± 0.09
Maximum strength [MPa]	418.60 ± 5.32	313.41 ± 4.95
$\langle R_g \rangle$ [Å]	20.19 (22.21)	14.25 (13.08)
$\langle R_{EE} \rangle$ [Å]	49.94 (54.99)	30.15 (31.19)

Table S.I: Initial structural and mechanical properties for bulk polyimide structures. R_g and R_{EE} values in the parenthesis are from the finite slab of polyimides.

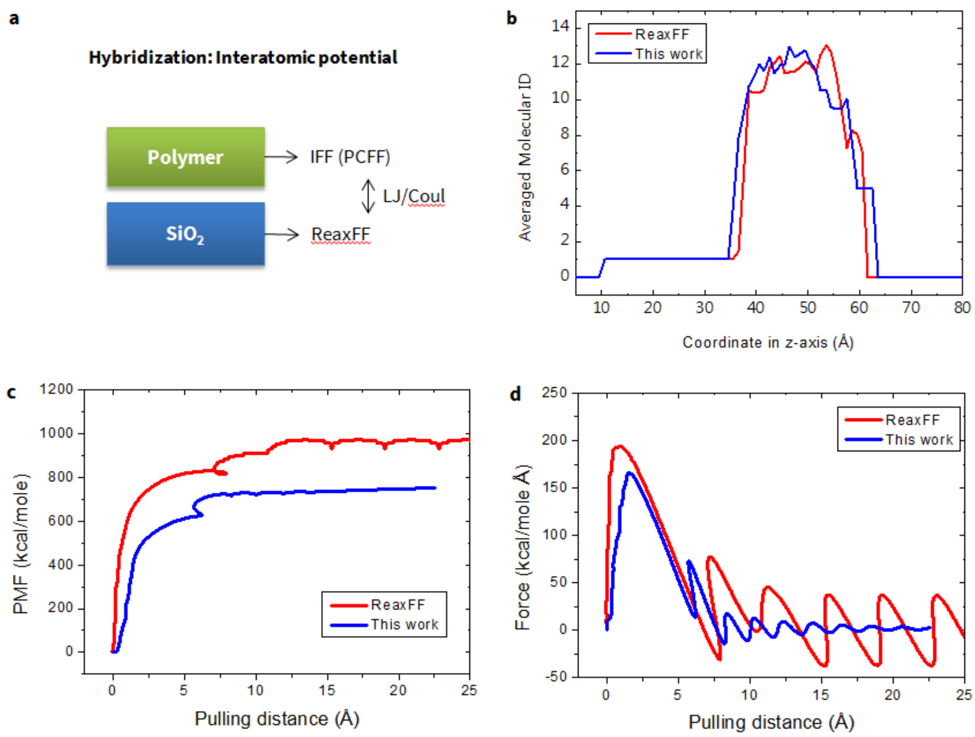

Figure S2. (a) The schematic view of hybridization of interatomic potentials. (b) The initial chain distribution, (c) PMF vs. pulling distance, and (d) pulling force vs. distance for comparison between ReaxFF and hybridized FF.

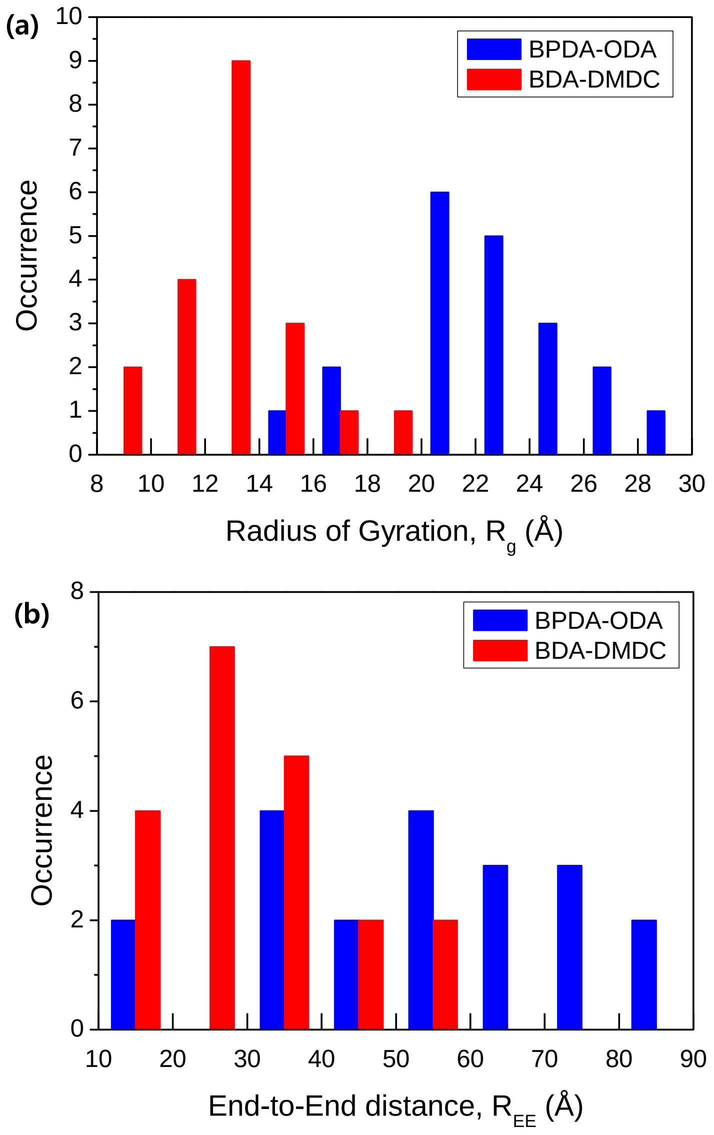

Figure S3. (a) R_g and (b) R_{EE} distribution for finite type of polyimide slabs.

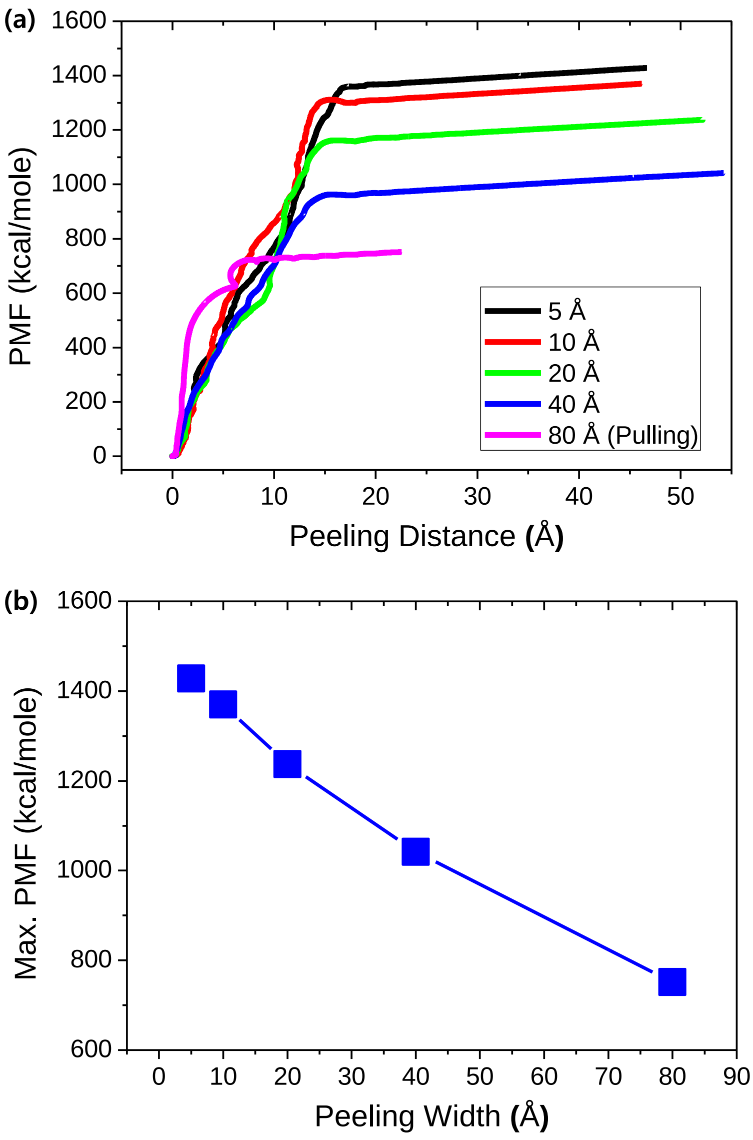

Figure S4. (a) PMF vs. peeling distance and (b) maximum PMF change as a function of peeling width

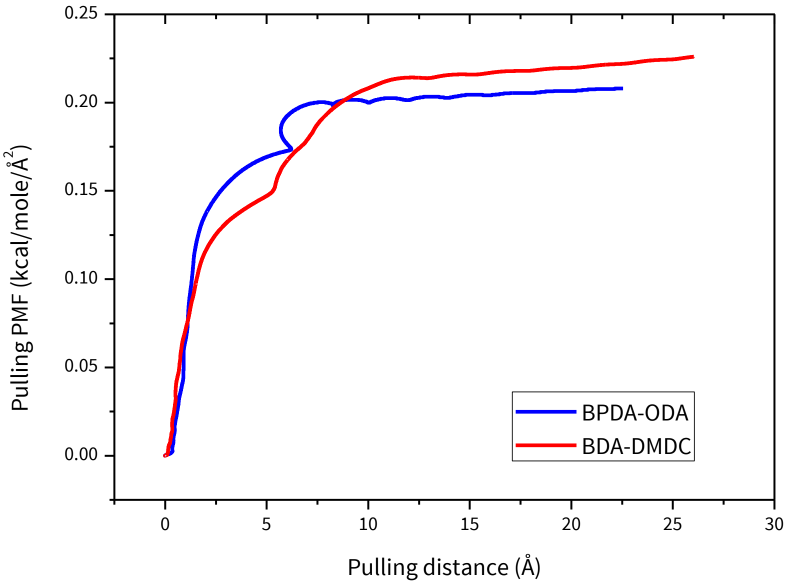

Figure S5. PMF vs. distance during puling test for both of PIs
